# Supplementary material for: Specific Biomarkers in Spinocerebellar Ataxia Type 3: A Systematic Review of Their Potential Uses in Disease Staging and Treatment Assessment
Source: Int J Mol Sci. 2024 Jul 24;25(15):8074. doi: 10.3390/ijms25158074 (PMC11311810; doi:10.3390/ijms25158074)
Supplement: Supplementary file 1 [file ijms-25-08074-s001.zip › ijms-3062618-supplementary.pdf]

International Journal of *Molecular Sciences*

MDPI

Review

Specific Biomarkers in Spinocerebellar Ataxia Type 3: A Systematic Review of Their Potential Uses in Disease Staging and Treatment Assessment

Alexandra E. Soto-Piña [1,2], Caroline C. Pulido-Alvarado [1], Jaroslaw Dulski [3,4,5], Zbigniew K. Wszolek [3,*] and Jonathan J. Magaña [6,7]

[1] Facultad de Medicina, Universidad Autónoma del Estado de México, Toluca 50180, Mexico; aesotop@uaemex.mx (A.E.S.-P.); carolpulidoalvarado@gmail.com (C.C.P.-A.)
[2] Department of Neuroscience, Mayo Clinic, Jacksonville, FL 32224, USA
[3] Department of Neurology, Mayo Clinic, Jacksonville, FL 32224, USA; jaroslaw.dulski@gumed.edu.pl
[4] Division of Neurological and Psychiatric Nursing, Faculty of Health Sciences Medical University of Gdansk, 80-211 Gdansk, Poland
[5] Neurology Department, St Adalbert Hospital, Copernicus PL Ltd., 80-462 Gdansk, Poland
[6] Department of Genomic Medicine, Instituto Nacional de Rehabilitación—Luis Guillermo Ibarra, Ibarra, Ciudad de México 14389, Mexico; jmagana@inr.gob.mx
[7] Department of Bioengineering, School of Engineering and Sciences, Tecnológico de Monterrey, Campus Ciudad de México, Ciudad de México 14380, Mexico
* Correspondence: wszolek.zbigniew@mayo.edu; Tel.: +1-904-953-7229

1. Supplementary material

Supplementary Table S1: Meta-analysis estimating mean values of NfL in controls, preclinical subjects, and ataxic subjects, separately for serum, plasma, and CSF.

	Controls			Preclinical subjects			Ataxic subjects		
Outcome	N	Mean (95% CI)	I2	N	Mean (95% CI)	I2	N	Mean (95% CI)	I2
NfL									
Serum	394	10.49 (6.21, 14.76)	96.0%	68	25.20 (13.99, 36.41)	87.7%	410	45.59 (26.53, 64.65)	97.0%
Plasma	68	8.31 (2.93, 13.69)	94.0%	15	16.75 (13.73, 19.76)	0.0%	67	29.36 (26.86, 31.86)	0.0%
CSF	43	458.40 (369.95, 546.85)	0.0%	N/A	N/A	N/A	63	3812.58 (3164.10, 4461.05)	53.6%

CI=confidence interval; Means and 95% CIs result from a random effect meta-analysis for continuous outcome variables.

Supplementary Table S2: Meta-analysis comparing NfL between controls, pre-clinical subjects, and ataxic subjects, separately for serum, plasma, and CSF.

	Preclinical subjects vs. controls			Ataxic subjects vs. controls			Ataxic vs. Preclinical subjects		
Outcome	Difference in means (95% CI)	p-value	I2	Difference in means (95% CI)	P-value	I2	Difference in means (95% CI)	p-value	I2
NfL									
Serum	13.50 (.76, 21.23)	0.0006	85.4%	34.94 (20.37, 49.51)	<0.0001	94.7%	18.22 (9.23, 27.21)	<0.0001	83.5%
Plasma	8.37 (0.46, 16.29)	0.038	82.7%	21.41 (17.26, 25.57)	<0.0001	53.0%	12.58 (8.64, 16.51)	<0.0001	0.0%
CSF	N/A	N/A	N/A	3347.66 (2725.48, 3969.85)	<0.0001	48.6%	N/A	N/A	N/A

CI=confidence interval. Differences in means, 95% CIs, and p-values result from a random effect meta-analysis for continuous outcome variables.

Supplementary Table S3: Potential trait and state biomarkers (discrimination of clinical stage and comorbidities).

Biomarker	Fluid	Control	Preataxic	Ataxic	Associated variables	Reference
t-tau	Plasma	--	Preataxic 30 years old: 0.42 [0.07; 0.77] log pg/mL higher than controls Preataxic > 300 pg/mL (controls cut-off)	Ataxic 30 years old: 0.42 [0.10; 0.73] log pg/mL higher than controls. Ataxic 40 years old: 0.29 [0.07; 0.51] log pg/mL higher than controls.	Age. Predictors: age and sex, explain 42.91% variability in preataxic, and 16.20% in ataxic. 0.51 log pg/mL increase in females. CAG repeats and higher INAS. T-tau levels decrease with age.	[1]
	CSF		384 [309; 464] pg/mL	227 [147; 322] pg/mL	None.	
	CSF	(104.6 ± 65.5 pg/mL)	--	106.3 ± 70.3 pg/mL)	Higher in AD than SCA3 patients. No difference between SCA3 and controls. No correlation with clinical variables	[2]
p-tau^{181}	CSF	--	(51 [44; 63] pg/mL)	32.5 [14; 41] pg/mL	Shorter disease duration. Relates to higher t-tau levels in CSF.	[1]
		--	--	29.9 ± 9.9 pg/mL	Higher in AD than SCA3. No difference between SCA3 and controls.	[2]
$A\beta_{42}$	CSF	(1138.4±121.0 pg/mL)	--	1272.1 ± 246.7 pg/mL	Levels in ataxic (1272.1 ± 246.7 pg/mL) are higher than AD (665.2 ± 330.7 pg/mL) and FTD	[2]

					(1055.1 ± 286.0 pg/mL) patients.	
GFAP	Plasma	--	-0.45 [-0.71; -0.21] log pg/mL lower than controls.	-0.20 [-0.33; -0.06] log pg/mL lower than controls.	Lower in ataxic and preataxic carriers (>30 years old) vs controls after age and sex adjustment. Increase with age.	[1]
	Blood	3.93±2.38 1.56–8.77	--	8.86±4.33 3.40–20.06	GFAP/age correlates with CAG repeat number.	[3]
UCHL1	Plasma	--	Lower in preataxic carriers vs controls,	No difference between ataxic and controls.	None.	[1]
pNfH	Serum	--	No differences between preataxic and controls.	--	pNfH in preataxic stage is not significantly lower than ataxic and were not significantly increased from controls. None.	[4]
S100B	Serum	--	--	No difference between controls and ataxic patients.	Moderate correlation with disease duration and depression.	[5]
	Serum	0.05 ± 0.02 ng/mL	--	0.07 ± 0.06 ng/mL	None.	[6]
NSE	Serum	4.65 ± 1.80 ng/mL	--	8.05 ± 4.20 ng/ml	Moderate correlation with depression and strong negative correlation with EDSS score.	[5]
	Serum	4.83 ± 1.70 ng/mL)	--	6.95 ± 2.8 ng/mL)	Positive correlation with age, disease duration, ICARS and SARA scores.	[6]

CHIP	Serum	(40.37 ± 18.55 ng/ml).	--	80.93 ± 28.68 ng/ml	Significantly associated with SARA and ICARS. Moderately correlated with age.	[7]
	CSF	37.47 ± 7.85 ng/ml		164.59 ± 42.99 ng/ml) vs controls	None.	
ROS (2′, 7′dichloro-fluorescein diacetate)	Serum	--	--	Increases in symptomatic patients (vs controls)	None.	[8]
SOD activity	Serum	--	--	Decreases in symptomatic patients vs presymptomatic	None.	[8]
GSH-PX activity	Serum	--	--	Decreases in symptomatic patients (vs controls).	Inverse correlation with NESSCA.	[8]
Thiol groups	Plasma	0.275 ± 0.047 nmol/mL).	--	0.112 ± 0.032 nmol/mL) than controls.	None.	[9]
Catalase activity	Whole blood	27.68 ± 10.02 mol/ H2O2 mL of erythrocytes/min	--	40.69 ± 10.09 mol/ H2O2mL of erythrocytes/min	None.	[9]
Eotaxin	Serum	Lower than asymptomatic	Higher than control and ataxic	Lower than preataxic	None.	[10]
L-proline	Serum	--	--	0.4 fold change vs controls and	Positively correlated with MMSE score.	[11]

				0.7 vs preataxic.		
FFA 16:0	Serum	--	--	0.4 fold change vs controls	Negatively related to ICARS.	[11]
Glucose	Serum	(63.3 ± 6.5 mg/dL).	--	92.2 ± 23.1 mg/dL	None	[9]
Triglycerides	Serum	94.6 ± 33.2 mg/dL).	--	(252.8 ± 120.9 mg/dL)	None.	[9]
Insulin	Serum	--	--	Decreases in SCA3 patients (corrected for body mass index).	Age at onset correlates with insulin levels.	[12]
IGFBP-1	Serum	--	--	Increases in ataxic patients.	Correlation with CAG expansion repeats.	[12]
IGFBP-3	Serum	--	--	Decreases in ataxic patients.	None.	[12]
IGF-1	Serum	--	--	No differences withcontrol patients.	None.	[12]
mir-7014	Plasma	--	--	Downregulated vs controls.	Correlation with age of onset.<br>Target gene pathways: protein processing in reticulum endoplasmic and axon guidance.	[13]
Mir-34b, mir-29 a, mir-25, mir-125b	Serum	--	--	Upregulated vs controls.	None.	[14]
m.8482_13460del4977 del	Blood	--	3X higher than controls	2X higher than controls.		[15]
SFSWAP	Blood	--	Increase vs controls	--	None.	[16]

SAFB2	Blood	--	Increase vs controls	Increase	Correlate with early age at onset.	[16]
LTBP4	Blood	--	Decrease vs controls	--	None.	[16]

Abbreviations: $A\beta_{42}$ = β-amyloid protein at amino acid 42, CSF = Cerebrospinal Fluid , AD = Alzheimer's disease, INAS = Inventory for Non-Ataxia Signs, GFAP = Glial fibrillary acidic protein, UCHL1 = Ubiquitin carboxy-terminal hydrolase, pNfH = Phosphorylated heavy chain neurofilament, IGF-1 = Insulin Grown Factor 1 and IGFBPs = IGF biding proteins, NSE = Neuron-specific enolase, CHIP = Carboxyl terminus of Hsp-70 protein, ROS = Reactive oxidative species, SOD = superoxide dismutase, GSH-Px = Glutathione peroxidase, GCDCA = glycochenodeoxycholate, , MMSE = Mini-Mental State Examination, ICARS = International Cooperative Ataxia Rating Scale, SARA = Scale for the Assessment and Rating of Ataxia, NESSCA = Neurological Examination Score for Spinocerebellar Ataxia, EDSS = Extended Disability Status Scale of Kurtzke, FTP = Frontotemporal dementia, FFA = Free Fatty Acid, SFSWAP: Splicing factor SWAP gene, SAFB2: Scaffold attachment factor B2, LTBP4: Latent transforming growth factor beta binding protein 4.

Supplementary Table S4. Current therapeutic strategies for SCA3.

Therapeutical Approach	Mechanisms	Development Stage	Sponsor	Reference
Riluzole	Riluzole is a N-methyl-D-aspartate (NMDA) receptor antagonist, reduces extracellular glutamate concentrations by inhibiting glutamate release	Unknown		[17-19]
Troriluzole	A prodrug of riluzole, troriluzole, was given for 96 weeks and reduced SARA scores indicating a potential benefit in SCA3 patients besides other types of ataxia	Phase 3	Biohaven, Inc.	[20]
Valproic Acid	VPA is a pan-HDAC inhibitor used clinically to treat bipolar and seizure disorders. VPA has proven an increase in SARA scores in SCA3	Randomized, double-blind, placebo-controlled, dose-controlled study		[21]
Trehalose	Disaccharide with protein-stabilizing and autophagy-enhancing properties	Phase 2a	Company name is missing	[22, 23]
ASOS	Target specific RNA molecules to modulate their function via degradation or modification of	VSO659 Phase 1/2a Trial	VICO Therapeutics	[24]

	translation and decreasing the adverse effects of the toxic protein		
CRISPR/Cas9	Deletion of the expanded polyQ-encoding region of ATXN3	No Clinical Trials	[25-27]
Stem Cell Therapy	Stem Cell Injection exert their reparative effects through secreting a broad repertoire of trophic factors	Preclinical	[28-30]

Abbreviations: ASOS: Antisense Oligonucleotides.

References:

1. Garcia-Moreno, H.; Prudencio, M.; Thomas-Black, G.; Solanky, N.; Jansen-West, K. R.; AL-Shaikh, R. H.; Heslegrave, A.; Zetterberg, H.; Santana, M. M.; de Almeida, L. P.; Vasconcelos-Ferreira, A.; Januario, C.; Infante, J.; Faber, J.; Klockgether, T.; Reetz, K.; Raposo, M.; Ferreira, A. F.; Lima, M.; Schols, L.; Synofzik, M.; Hubener-Schmid, J.; Puschmann, A.; Gorcenco, S.; Wszolek, Z. K.; Petrucelli, L.; Giunti, P., Tau and neurofilament light-chain as fluid biomarkers in spinocerebellar ataxia type 3. European Journal of Neurology 2022, 29, (8), 2439-2452.
2. Ye, L. Q.; Li, X. Y.; Zhang, Y. B.; Cheng, H. R.; Ma, Y.; Chen, D. F.; Tao, Q. Q.; Li, H. L.; Wu, Z. Y., The discriminative capacity of CSF beta-amyloid 42 and Tau in neurodegenerative diseases in the Chinese population. J Neurol Sci 2020, 412, 116756.
3. Shi, Y.; Wang, C.; Huang, F.; Chen, Z.; Sun, Z.; Wang, J.; Tang, B.; Ashizawa, T.; Klockgether, T.; Jiang, H., High Serum GFAP Levels in SCA3/MJD May Not Correlate with Disease Progression. Cerebellum 2015, 14, (6), 677-81.
4. Wilke, C.; Haas, E.; Reetz, K.; Faber, J.; Garcia-Moreno, H.; Santana, M. M.; van de Warrenburg, B.; Hengel, H.; Lima, M.; Filla, A.; Durr, A.; Melegh, B.; Masciullo, M.; Infante, J.; Giunti, P.; Neumann, M.; de Vries, J.; Pereira de Almeida, L.; Rakowicz, M.; Jacobi, H.; Schule, R.; Kaeser, S. A.; Kuhle, J.; Klockgether, T.; Schols, L.; group, S. C. A. n. s.; Barro, C.; Hubener-Schmid, J.; Synofzik, M., Neurofilaments in spinocerebellar ataxia type 3: blood biomarkers at the preataxic and ataxic stage in humans and mice. EMBO Mol Med 2020, 12, (7), e11803.
5. Tort, A. B.; Portela, L. V.; Rockenbach, I. C.; Monte, T. L.; Pereira, M. L.; Souza, D. O.; Rieder, C. R.; Jardim, L. B., S100B and NSE serum concentrations in Machado Joseph disease. Clin Chim Acta 2005, 351, (1-2), 143-8.
6. Zhou, J.; Lei, L.; Shi, Y.; Wang, J.; Jiang, H.; Shen, L.; Tang, B., Serum concentrations of NSE and S100B in spinocerebellar ataxia type 3/Machado-Joseph disease. Zhong Nan Da Xue Xue Bao Yi Xue Ban 2011, 36, (6), 504-10.
7. Hu, Z. W.; Yang, Z. H.; Zhang, S.; Liu, Y. T.; Yang, J.; Wang, Y. L.; Mao, C. Y.; Zhang, Q. M.; Shi, C. H.; Xu, Y. M., Carboxyl Terminus of Hsp70-Interacting Protein Is Increased in Serum and Cerebrospinal Fluid of Patients With Spinocerebellar Ataxia Type 3. Front Neurol 2019, 10, 1094.
8. de Assis, A. M.; Saute, J. A. M.; Longoni, A.; Haas, C. B.; Torrez, V. R.; Brochier, A. W.; Souza, G. N.; Furtado, G. V.; Gheno, T. C.; Russo, A.; Monte, T. L.; Castilhos, R. M.; Schumacher-Schuh, A.; D'Avila, R.; Donis, K. C.; de Mello Rieder, C. R.; Souza, D. O.; Camey, S.; Leotti, V. B.; Jardim, L. B.; Portela, L. V., Peripheral Oxidative Stress Biomarkers in Spinocerebellar Ataxia Type 3/Machado-Joseph Disease. Front Neurol 2017, 8, 485.
9. Pacheco, L. S.; da Silveira, A. F.; Trott, A.; Houenou, L. J.; Algarve, T. D.; Bello, C.; Lenz, A. F.; Manica-Cattani, M. F.; da Cruz, I. B. M., Association between Machado-Joseph disease and oxidative stress biomarkers. Mutation Research-Genetic Toxicology and Environmental Mutagenesis 2013, 757, (2), 99-103.
10. da Silva Carvalho, G.; Saute, J. A.; Haas, C. B.; Torrez, V. R.; Brochier, A. W.; Souza, G. N.; Furtado, G. V.; Gheno, T.; Russo, A.; Monte, T. L.; Schumacher-Schuh, A.; D'Avila, R.; Donis, K. C.; Castilhos, R. M.; Souza, D. O.; Saraiva-

Pereira, M. L.; Torman, V. L.; Camey, S.; Portela, L. V.; Jardim, L. B., Cytokines in Machado Joseph Disease/Spinocerebellar Ataxia 3. Cerebellum 2016, 15, (4), 518-25.
11. Yang, Z. H.; Shi, C. H.; Zhou, L. N.; Li, Y. S.; Yang, J.; Liu, Y. T.; Mao, C. Y.; Luo, H. Y.; Xu, G. W.; Xu, Y. M., Metabolic Profiling Reveals Biochemical Pathways and Potential Biomarkers of Spinocerebellar Ataxia 3. Front Mol Neurosci 2019, 12, 159.
12. Saute, J. A.; da Silva, A. C.; Muller, A. P.; Hansel, G.; de Mello, A. S.; Maeda, F.; Vedolin, L.; Saraiva-Pereira, M. L.; Souza, D. O.; Arpa, J.; Torres-Aleman, I.; Portela, L. V.; Jardim, L. B., Serum insulin-like system alterations in patients with spinocerebellar ataxia type 3. Mov Disord 2011, 26, (4), 731-5.
13. Hou, X.; Gong, X.; Zhang, L.; Li, T.; Yuan, H.; Xie, Y.; Peng, Y.; Qiu, R.; Xia, K.; Tang, B.; Jiang, H., Identification of a potential exosomal biomarker in spinocerebellar ataxia Type 3/Machado-Joseph disease. Epigenomics 2019, 11, (9), 1037-1056.
14. Shi, Y.; Huang, F.; Tang, B.; Li, J.; Wang, J.; Shen, L.; Xia, K.; Jiang, H., MicroRNA profiling in the serums of SCA3/MJD patients. Int J Neurosci 2014, 124, (2), 97-101.
15. Raposo, M.; Ramos, A.; Santos, C.; Kazachkova, N.; Teixeira, B.; Bettencourt, C.; Lima, M., Accumulation of Mitochondrial DNA Common Deletion Since The Preataxic Stage of Machado-Joseph Disease. Mol Neurobiol 2019, 56, (1), 119-124.
16. Raposo, M.; Hubener-Schmid, J.; Ferreira, A. F.; Vieira Melo, A. R.; Vasconcelos, J.; Pires, P.; Kay, T.; Garcia-Moreno, H.; Giunti, P.; Santana, M. M.; Pereira de Almeida, L.; Infante, J.; van de Warrenburg, B. P.; de Vries, J. J.; Faber, J.; Klockgether, T.; Casadei, N.; Admard, J.; Schols, L.; European Spinocerebellar ataxia type 3/Machado-Joseph disease Initiative study, g.; Riess, O.; Lima, M., Blood transcriptome sequencing identifies biomarkers able to track disease stages in spinocerebellar ataxia type 3. Brain 2023.
17. Dulski, J.; Al-Shaikh, R. H.; Sulek, A.; Kasprzak, J.; Slawek, J.; Wszolek, Z. K., Spinocerebellar ataxia type 3 (Machado-Joseph disease). Pol Arch Intern Med 2022, 132, (10).
18. Ristori, G.; Romano, S.; Visconti, A.; Cannoni, S.; Spadaro, M.; Frontali, M.; Pontieri, F. E.; Vanacore, N.; Salvetti, M., Riluzole in cerebellar ataxia A randomized, double-blind, placebo-controlled pilot trial. Neurology 2010, 74, (10), 839-845.
19. Romano, S.; Coarelli, G.; Marcotulli, C.; Leonardi, L.; Piccolo, F.; Spadaro, M.; Frontali, M.; Ferraldeschi, M.; Vulpiani, M. C.; Ponzelli, F.; Salvetti, M.; Orzi, F.; Petrucci, A.; Vanacore, N.; Casali, C.; Ristori, G., Riluzole in patients with hereditary cerebellar ataxia: a randomised, double-blind, placebo-controlled trial. Lancet Neurology 2015, 14, (10), 985-991.
20. Wirtz, V.; L'Italien, G.; Berman, R.; Beiner, M., Results from the Long-Term Open Label Extension Phase Analyses of BHV4157-201: A Phase IIb/III, Randomized, Double-blind, Placebo-controlled Trial of the Safety and Efficacy of Troriluzole in Adult Subjects with Spinocerebellar Ataxia. Neurology 2020, 94, (15).
21. Lei, L. F.; Yang, G. P.; Wang, J. L.; Chuang, D. M.; Song, W. H.; Tang, B. S.; Jiang, H., Safety and efficacy of valproic acid treatment in SCA3/MJD patients. Parkinsonism Relat Disord 2016, 26, 55-61.
22. Noorasyikin, M. A.; Azizan, E. A.; Teh, P. C.; Waheeda, T. F.; Hajar, M. D. S.; Long, K. C.; Norlinah, M. I., Oral trehalose maybe helpful for patients with spinocerebellar ataxia 3 and should be better evaluated. Parkinsonism & Related Disorders 2020, 70, 42-44.
23. Zaltzman, R.; Elyoseph, Z.; Lev, N.; Gordon, C. R., Trehalose in Machado-Joseph Disease: Safety, Tolerability, and Efficacy. Cerebellum 2020, 19, (5), 672-679.
24. Clinicaltrials.gov. Section: Spinocerebellar Ataxia Type 3 - BIIB132 Available online https://clinicaltrials.gov/study/NCT05160558?cond=sca3&viewType=Table&page=2&rank=12 (accessed on April 24th 2023).

25. Babacic, H.; Mehta, A.; Merkel, O.; Schoser, B., CRISPR-cas gene-editing as plausible treatment of neuromuscular and nucleotide-repeat-expansion diseases: A systematic review. Plos One 2019, 14, (2).
26. He, L.; Wang, S.; Peng, L.; Zhao, H.; Li, S.; Han, X.; Habimana, J. D.; Chen, Z.; Wang, C.; Peng, Y.; Peng, H.; Xie, Y.; Lei, L.; Deng, Q.; Wan, L.; Wan, N.; Yuan, H.; Gong, Y.; Zou, G.; Li, Z.; Tang, B.; Jiang, H., CRISPR/Cas9 mediated gene correction ameliorates abnormal phenotypes in spinocerebellar ataxia type 3 patient-derived induced pluripotent stem cells. Transl Psychiatry 2021, 11, (1), 479.
27. Ouyang, S.; Xie, Y.; Xiong, Z.; Yang, Y.; Xian, Y.; Ou, Z.; Song, B.; Chen, Y.; Xie, Y.; Li, H.; Sun, X., CRISPR/Cas9-Targeted Deletion of Polyglutamine in Spinocerebellar Ataxia Type 3-Derived Induced Pluripotent Stem Cells. Stem Cells Dev 2018, 27, (11), 756-770.
28. Correia, J. S.; Duarte-Silva, S.; Salgado, A. J.; Maciel, P., Cell-based therapeutic strategies for treatment of spino-cerebellar ataxias: an update. Neural Regen Res 2023, 18, (6), 1203-1212.
29. Joyce, N.; Annett, G.; Wirthlin, L.; Olson, S.; Bauer, G.; Nolta, J. A., Mesenchymal stem cells for the treatment of neurodegenerative disease. Regen Med 2010, 5, (6), 933-46.
30. Paul, G.; Anisimov, S. V., The secretome of mesenchymal stem cells: potential implications for neuroregeneration. Biochimie 2013, 95, (12), 2246-56.
